# Dermoscopic features in children with vitiligo and other hypopigmentation disorders

**DOI:** 10.3389/fped.2025.1550349

**Published:** 2025-07-10

**Authors:** Shijuan Yu, Jingyi He, Hua Wang

**Affiliations:** ^1^ Department of Dermatology, National Clinical Research Center for Child Health and Disorders, Ministry of Education Key Laboratory of Child Development and Disorders, China International Science and Technology Cooperation Base of Child Development and Critical Disorders, Chongqing Key Laboratory of Child Infection and Immunity, Children’s Hospital of Chongqing Medical University, Chongqing, China; ^2^ Department of Dermatology, The Seventh People’s Hospital of Chongqing/The Central Hospital Affiliated to Chongqing University of Technology, Chongqing, China

**Keywords:** dermatoscope, vitiligo, differential diagnosis, evaluation, depigmentation

## Abstract

**Objective:**

To analyze the application value of dermoscopy in the identification and staging evaluation of vitiligo in children.

**Methods:**

We have analyzed the differences in dermoscopy between vitiligo and other hypopigmentation disorders. Meanwhile, the dermatoscopic differences between active and stable vitiligo were also compared. At the same time, the results of diagnosing vitiligo were compared between a single dermascopic image (testing paper) and a combination of photographs, Wood's lamp examinations, and dermascopic images (training paper) by answering a questionnaire to eight dermatologists.

**Results:**

We have summarized the dermatoscopic features and clinical characteristics of 112 cases of active vitiligo, 111 cases of stable vitiligo, 99 cases of pityriasis alba, 94 cases of depigmented nevi, 16 cases of sclerosing atrophic moss, and 42 cases of post-inflammatory hypopigmentation in children. In this study, a statistically significant difference (*p* < 0.05) was found in the dermatoscopic characteristics between vitiligo at different stages and several other pigmentary disorders. Meanwhile, the trichrome pattern at the periphery of the lesion [area under the curve (AUC) = 0.8834, sensitivity 89.19%, specificity 87.5%] and the micro-Koebner/comet tail phenomenon (AUC = 0.7812, sensitivity 99.1%, specificity 57.14%) showed better diagnostic efficacy for active vitiligo, while the pigmentation at the periphery of the lesion (AUC = 0.8746, sensitivity 91.89%, specificity 83.04%) showed better diagnostic efficacy for stable vitiligo. The mean score (79.75 ± 10.45 vs. 71 ± 3.85; *p* = 0.058) and median time [555.50 (705) vs. 374 (1,011) s; *p* = 1,000] for physicians completing training and testing papers showed no statistical difference.

**Conclusion:**

Dermatoscopy can serve as a standalone tool for diagnosing vitiligo in children and may also help in staging the condition.

## 1 Introduction

Vitiligo is a chronic and progressive autoimmune skin disease, characterized by the loss of function of melanocytes in the skin or mucous membranes (1). It often has significant cosmetic impairment and brings considerable psychological burden to patients (2). Due to the complex mechanisms of vitiligo, which involves multiple factors such as genetics, autoimmunity, oxidative stress, and environment (3), early diagnosis and management may improve clinical efficacy and prognosis by blocking vicious progression as early as possible (4). However, the diagnosis of vitiligo still largely depends on the clinical experience of physicians, Wood's lamp examination, or even a skin biopsy in some cases (5). These methods can be highly subjective and are often influenced by confounding factors (6). Dermoscopy is a sub-macroscopic diagnostic technique that uses optical magnification and polarized light to clearly observe subtle structures, such as pigments and blood vessels, in the epidermis and dermis that may not be visible to the naked eye (7). It holds significant value for the early assessment of dermatologic conditions (8). To date, dermatoscopy research has mostly focused on pigmented nevi, melanoma, basal cell carcinoma, hemangioma, and other hyperpigmented lesions or skin tumors (9–11). There are relatively few studies on depigmented disorders, such as vitiligo (12–14). Therefore, this study aimed to analyze the dermoscopic features of vitiligo in comparison with other hypopigmentary disorders and to evaluate dermatologists’ diagnostic accuracy using dermoscopic images, thereby assessing its clinical utility in identifying vitiligo.

## 2 Materials and methods

### 2.1 Study participants

Data were collected from a specialized dermatology clinic for vitiligo in a tertiary teaching children's hospital in southwestern China between 9 October 2022 and 1 August 2023. Vitiligo and other common depigmented diseases (including pityriasis albuginea, depigmented nevi, sclerosing atrophic moss, and post-inflammatory hypopigmentation) were diagnosed by two dermatologists who held the title of at least attending doctor and with more than 10 years of experience. All enrolled patients signed informed consent forms. This study was approved by the hospital ethics committee [approval number: (2022) Lunshen (Research) No. (249)] and registered with the China Clinical Trial Registry (registration number: ChiCTR2200064339).

The inclusion criteria were as follows: all patients were aged under 18 years. For vitiligo, inclusion was based on the worldwide expert recommendations for the diagnosis and management of vitiligo (5), which incorporate a combination of the patient's medical history, clinical presentation, Wood's lamp examination, dermoscopic evaluation, and the exclusion of other autoimmune diseases. Disease staging was determined according to the following: the presence of new or expanding lesions within the past year, trichrome lesions, poorly defined borders, confetti-like depigmentation, inflammatory lesions, or Koebner's phenomenon were considered indicative of the active or progressive phase (15–17). The absence of these symptoms indicated the condition was considered stable. For other hypopigmentation disorders, inclusion required the presence of clinical hypopigmentation spots, supported by medical history, Wood's lamp and dermoscopy examinations, and histopathological and biochemical examinations where necessary (18–23).

Exclusion criteria included patients with systemic diseases, severe infections, lack of cooperation, or suspected (but not newly diagnosed) cases.

Imaging data included dermoscopy, Wood's lamp images, and standard photographs. Clinical data included patient age, gender, disease duration, lesion location, comorbidities, medical history, family history, history of trauma or exposure, vitiligo classification, Vitiligo Disease Activity Score (VIDA) (24), body surface area (BSA) affected, Vitiligo Area Score Index (VASI) (25), Children's Dermatology Life Quality Index (CDLQI) (26), and visual analog scale (VAS) (27).

All patients underwent dermatoscopy examination performed by the same staff member using the same device (specification model: CH-DSIS-2000 Plus, lens: optical magnification 50×, polarized light; Guangzhou Chuanghong Medical Technology Co., Ltd., Guangzhou, China). The evaluation of dermoscopic images was independently performed by two blinded physicians. The physicians responsible for dermatoscopic diagnosis had 5 years of experience in dermatoscopy (28–32) and held the “China Skin Imaging Technology Intermediate Ability Certification,” issued by the Talent Exchange Service Center of the National Health Commission.

### 2.2 Research methods

The technical route is shown in Figure S1 in the Supplementary Material. We compared the dermatoscopic features and clinical data of vitiligo at different stages with several other hypopigmentation diseases. Two sets of test papers were designed. First, 25 cases of vitiligo and 25 cases of other hypopigmentary diseases were randomly selected. Clinical photographs, Wood's lamp images, and dermoscopic images from each case were used to create a training set comprising 50 questions. Subsequently, another 25 cases of vitiligo and 25 cases of other hypopigmentary diseases were randomly selected, and only dermoscopic images from these cases were compiled into a test set of 50 questions. Eight dermatologists who had worked in tertiary hospitals for more than 5 years (without experience in diagnosing vitiligo by dermatoscopy) first independently completed the training paper (answers could be viewed for learning after each question was completed) and then independently completed the test paper. The assessments were conducted online.

### 2.3 Statistical analysis

A statistical analysis was conducted using SPSS version 26 (IBM Corp., Armonk, NY, USA), assuming that a two-sided *p*-value < 0.05 was statistically significant. The quantitative data of normal distribution was represented by the mean ± standard deviation, while variables of non-normal distribution were summarized as median and interquartile range (IQR). Categorical dermoscopic features were expressed as rates and composition ratios, and analyzed using the chi-square test. For non-independent categorical variables, such as dermoscopic features of the same patient before and after treatment, McNemar's test was applied. Paired *t*-tests and symbol rank sum test for paired design were used to compare training scores. Correlation between clinical variables and dermoscopic features was assessed using logistic regression analysis. All graphs were generated using GraphPad Prism (version 9.1.1).

## 3 Results

### 3.1 Clinical data and dermoscopic characteristics

**Figure 1:**
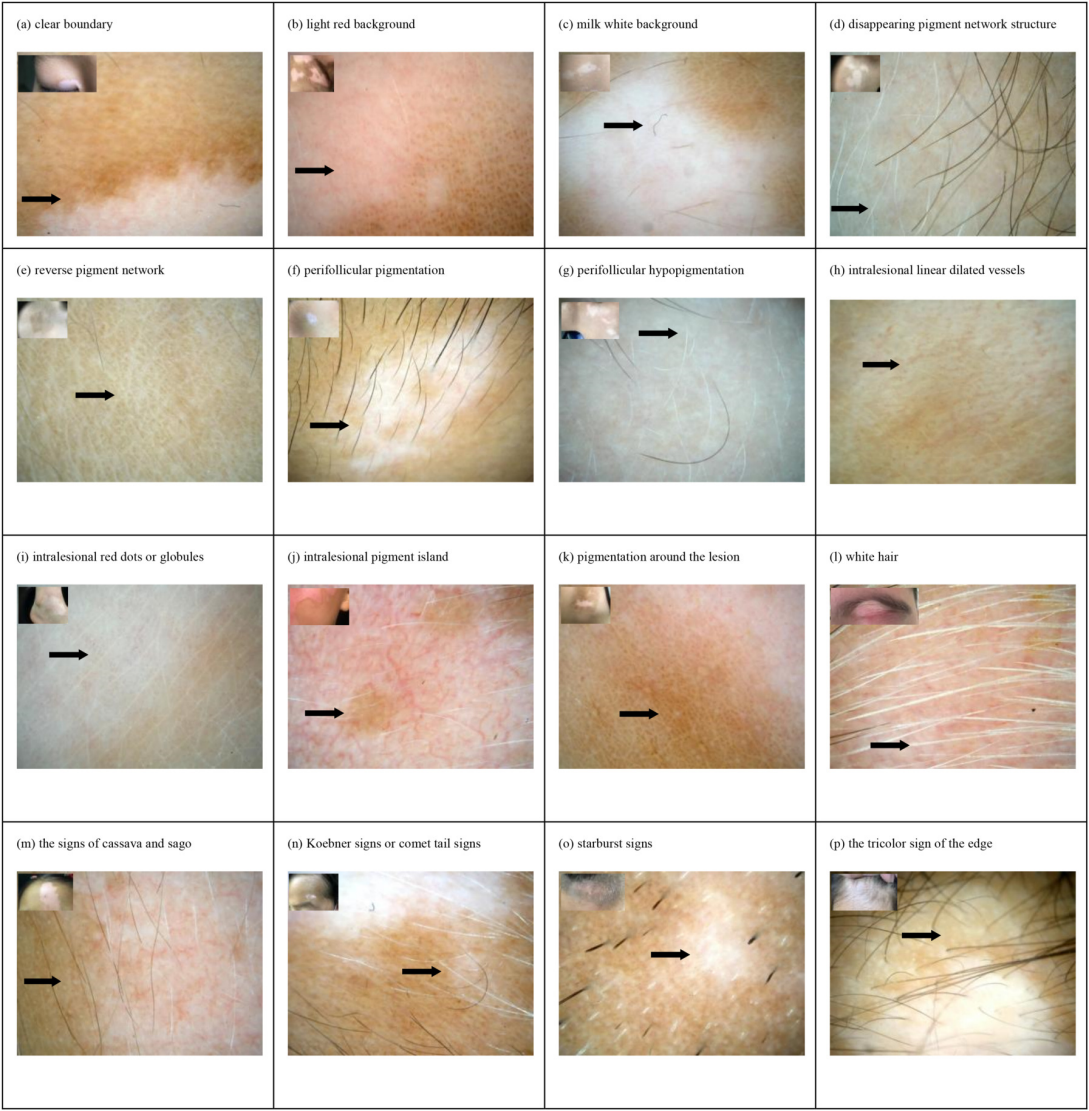
Dermoscopic features and clinical manifestations of vitiligo in children: (**a**) clear boundary, (**b**) light red background, (**c**) milk white background, (**d**) disappearing pigment network structure, (**e**) reverse pigment network, (**f**) perifollicular pigmentation, (**g**) perifollicular hypopigmentation, (**h**) intralesional linear dilated vessels, (**i**) intralesional red dots or globules, (**j**) intralesional pigment isle, (**k**) pigmentation around the lesion, (**l**) white hair, (**m**) the signs of cassava and sago, (**n**) Koebner signs or comet tail signs, (**o**) starburst signs, and (**p**) the tricolor sign of the edge.

This study collected clinical data and dermoscopic features of 112 cases of active vitiligo, 111 cases of stable or recovering vitiligo, 99 cases of pityriasis albuginea, 94 cases of depigmented nevi, 42 cases of post-inflammatory hypopigmentation, and 16 cases of sclerosing atrophic lichen (Table 1). Of the 112 cases of active vitiligo, 46 cases completed follow-up after treatment. The clinical data and dermoscopic features of the 46 cases before (active stage) and after treatment (stable or recovery stage) were summarized (see Tables S1, S2 in the Supplementary Material). The collected typical dermatoscopic features of vitiligo include distinct margins, chalky white background, intralesional erythema, reduced or absent pigment network, leukotrichia, perifollicular pigmentation, perifollicular depigmentation, telangiectasia, intralesional red dots of globules, intralesional repigmentation isles, perilesional/marginal hyperpigmentation, inverted pigment network, tapioca sago appearance, starburst pattern, micro-Koebner phenomenon, comet tail pattern, and trichromic pattern (Figure 1). The other atypical dermatoscopic features include scattered small pearly white globules, perifollicular depigmentation, follicular keratin plugs, perilesional erythema, tiny grayish-white scales, pale hypopigmentation spots, serrated edges, reticular distribution, irregular morphology, gray network, thin or atrophic epidermis, scar-like white structures, acne-like openings, and dotted or reticular pigmentation.

**Table 1:** Summary of clinical information and dermatoscopic features of the six diseases.

Variable	Subitems	Active vitiligo (*N* = 112)	Stable or recovering vitiligo (*N* = 111)	*p*-value
Sex	Male	59 (52.7%)	63 (56.8%)	0.591^a^
	Female	53 (47.3%)	48 (43.2%)	
Location	Head, face, and neck	47 (42%)	56 (50.5%)	0.090^a^
	Trunk	36 (32.1%)	31 (27.9%)	
	Limbs	13 (11.6%)	18 (16.2%)	
	≥2 region	16 (14.3%)	6 (5.4%)	
Dermatoscopic features	Clear boundary	2 (1.8%)	77 (69.4%)	0.000^a^
	Milk white background	111 (99.1%)	74 (66.7%)	0.000^a^
	Disappearing pigment network structure	111 (99.1%)	104 (93.7%)	0.070^b^
	Inverted pigment network	40 (35.7%)	49 (44.1%)	0.220^a^
	Perifollicular pigmentation	39 (34.8%)	55 (49.5%)	0.030^a^
	Perifollicular hypopigmentation	107 (95.5%)	95 (85.6%)	0.012^a^
	Intralesional telangiectasia	61 (54.5%)	100 (90.1%)	0.000^a^
	Intralesional red dots or globules	52 (46.4%)	74 (66.7%)	0.003^a^
	Intralesional repigmentation isles	25 (22.3%)	67 (60.4%)	0.000^a^
	Pigmentation around the lesion	19 (17%)	102 (91.9%)	0.000^a^
	Leukotrichia	60 (53.6%)	80 (72.1%)	0.006^a^
	Tapioca sago sign	36 (32.1%)	26 (23.4%)	0.179^a^
	Micro-Koebner phenomenon/comet tail pattern	64 (57.1%)	1 (0.9%)	0.000^a^
	Starburst signs	42 (37.5%)	6 (5.4%)	0.000^a^
	Trichromic pattern	98 (87.5%)	12 (10.8%)	0.000^a^
	Light red background	32 (28.6%)	76 (68.5%)	0.000^a^
Variable		Pityriasis alba (*N* = 99)		
Sex	Male	55 (55.6%)		
	Female	44 (44.4%)		
Location	Cheeks	92 (92.9%)		
	Forehead	6 (6.1%)		
	Limbs	1 (1%)		
Dermatoscopic features	Clear boundary	0		
	Scattered white dots	84 (84.8%)		
	White halo around hair follicles	96 (97%)		
	Follicular keratin plug	32 (32.3%)		
	Pale white or reddish patches at the edges of lesions	2 (2.0%)		
	Few fine grayish-white scales on the surface	88 (88.9%)		
	Linear dilated vessels	82 (82.8%)		
Variable		Nevus depigmentosus (*N* = 94)		
Sex	Male	51 (54.3%)		
	Female	43 (45.7%)		
Location	Face and neck	29 (30.9%)		
	Trunk	37 (39.4%)		
	Limbs	28 (29.8%)		
Dermatoscopic features	Clear boundary	11 (11.7%)		
	Pallid hypopigmented patches	94 (100%)		
	Serrated edge	80 (85.1%)		
	Reticulate distribution	30 (31.9%)		
	Irregular shape	93 (98.9%)		
	No marginal pigmentation	94 (100%)		
	No perifollicular hyperpigmentation	93 (98.9%)		
	Linear dilated vessels	74 (78.7%)		
Variable		Post-inflammatory hypopigmentation (*N* = 42)		
Sex	Male	24 (57.1%)		
	Female	18 (42.9%)		
Variable	Subitems	Post-inflammatory hypopigmentation		
Location	Head, face, neck	23 (54.8%)		
	Trunk	9 (21.4%)		
	Limbs	10 (23.8%)		
Dermatoscopic features	Clear boundary	6 (14.3%)		
	White background	42 (100%)		
	White network	22 (52.4%)		
	Epidermal thinning or atrophy	15 (35.7%)		
	Scales	40 (95.2%)		
	Perifollicular hypopigmentation	41 (97.6%)		
	Pigmentation around the lesion	17 (40.5%)		
	Intralesional red dots or globules	19 (45.2%)		
	Linear dilated vessels	23 (54.8%)		
Variable		Lichen sclerosus (*N* = 16)		
Sex	Male	0		
	Female	16 (100%)		
Location	External genitalia	15 (93.8%)		
	Non-external genitalia	1 (6.3%)		
Dermatoscopic features	Clear boundary	1 (6.3%)		
	White scar-like structure	16 (100%)		
	Pimple-like openings	14 (87.5%)		
	Dotted or reticular hyperpigmentation	16 (100%)		
	Linear dilated vessels	16 (100%)		

^a^ Chi-square test.

^b^ Adjusted chi-square test.

### 3.2 Analysis of dermoscopic characteristics

**Figure 2:**
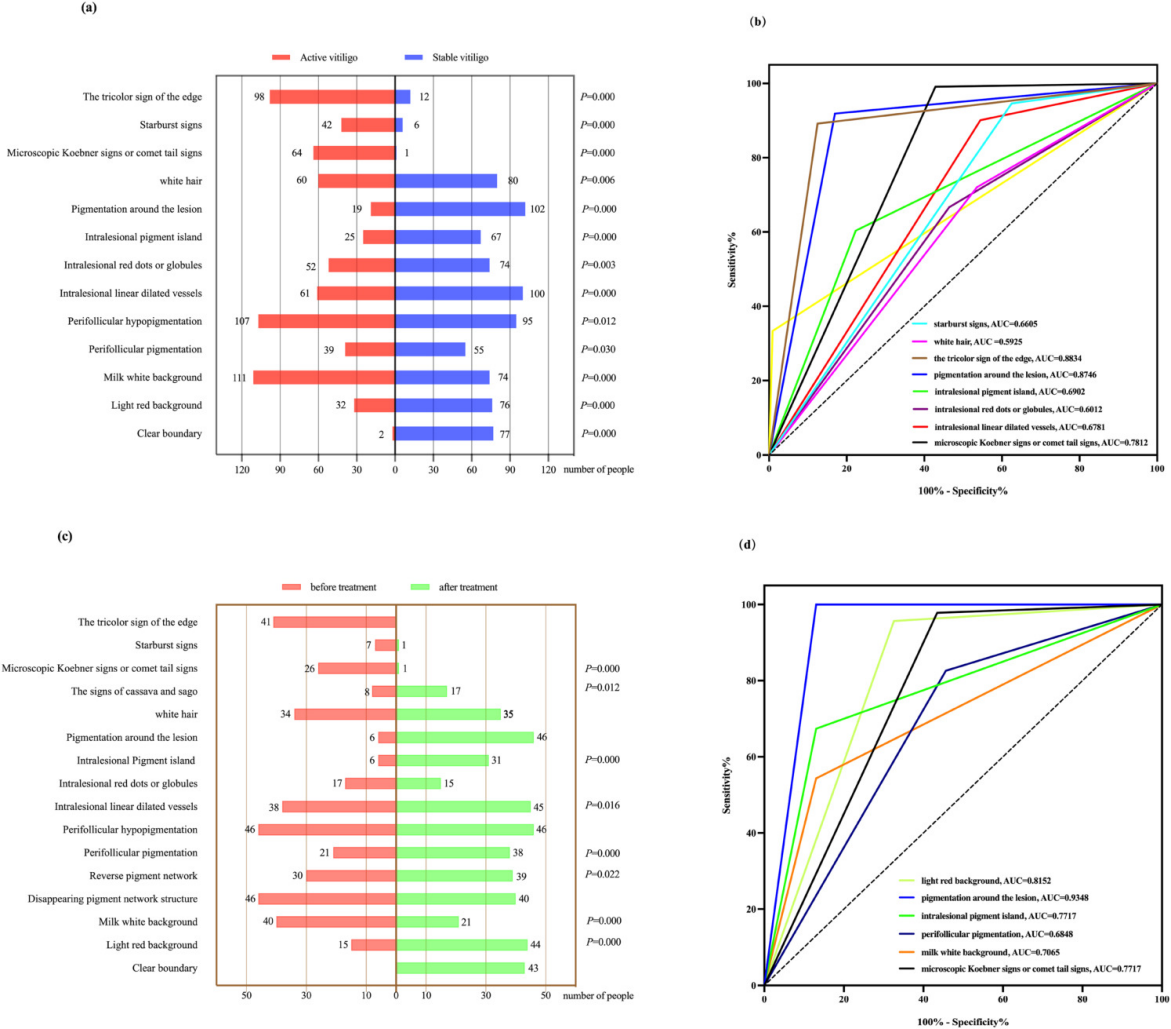
(**a,b**) Chi-square test results and ROC curves of dermoscopic features in 112 active vitiligo and 111 stable vitiligo cases. (**c,d**) McNemar's test results and ROC curves of dermoscopic features before (active) and after (stable) treatment in 46 active vitiligo cases. ROC, receiver operating characteristic.

The results showed that there were statistically significant differences (*p* < 0.05) in the dermatoscopic features of 112 cases of active vitiligo and 111 cases of stable vitiligo, including distinct margins, chalky white background, perifollicular pigmentation, perifollicular depigmentation, telangiectasia, intralesional red dots or globules, repigmentation isles, perilesional/marginal hyperpigmentation, leukotrichia, micro-Koebner phenomenon/comet tail pattern and trichromic pattern, starburst pattern, and intralesional erythema (Figure 2A). Among them, the trichromic pattern [area under the curve (AUC) = 0.8834, sensitivity 89.19%, specificity 87.5%] and the micro-Koebner phenomenon/comet tail pattern (AUC = 0.7812, sensitivity 99.1%, specificity 57.14%) had better diagnostic efficacy for active vitiligo, whereas perilesional hyperpigmentation (AUC = 0.8746, sensitivity 91.89%, specificity 83.04%) had better diagnostic efficacy for stable vitiligo (Figure 2B). Statistically significant differences (*p* < 0.05) were observed in dermoscopic features of the 46 active vitiligo cases before and after treatment. These included intralesional erythema, milky white background, inverted pigment network, perifollicular pigmentation, telangiectasia, intralesional repigmentation isles, tapioca sago sign, and the micro-Koebner phenomenon/comet tail pattern (Figure 2C). Among them, intralesional erythema (AUC = 0.8152, sensitivity 95.65%, specificity 67.39%), perifollicular depigmentation (AUC = 0.9348, sensitivity 100%, specificity 86.96%), and intralesional repigmentation isles (AUC = 0.7717, sensitivity 67.39%, specificity 86.96%) showed high diagnostic efficacy for identifying stable or recovering vitiligo. In contrast, the micro-Koebner phenomenon/comet tail pattern (AUC = 0.7717, sensitivity 97.83%, specificity 56.52%) showed high diagnostic efficacy for active stage vitiligo (Figure 2D).

Under dermatoscopy, pityriasis albuginea is characterized by scattered small pearly white globules (84.8%), perifollicular depigmentation (97%), and fine grayish-white scales (88.9%). Non-pigmented nevi present with serrated edges (85.1%) and pale hypopigmentation spots (100%). The characteristic features of post-inflammatory pigmentation include scales (95.2%), thin or atrophic epidermis (35.7%), and a gray pigment network (52.4%). The characteristic features of sclerosing atrophic moss include scar-like white structures (100%), acne-like openings (87.5%), and dotted or reticular pigmentation (100%) (Table 1). Pairwise comparisons using the chi-square test revealed statistically significant differences (*p* < 0.05) between white pityriasis and both active and stable vitiligo, as well as between depigmented nevi and stable vitiligo, in terms of different locations. In the analysis of dermoscopic features with distinct boundaries, statistical differences (*p* < 0.05) were observed between depigmented nevi and active/stable vitiligo, post-inflammatory hypopigmentation and active/stable vitiligo, sclerosing atrophic moss and stable vitiligo, and between active and stable vitiligo. In the analysis of dermatoscopic features related to telangiectasia, statistically significant differences (*p* < 0.05) were found between white pityriasis and active vitiligo, depigmented nevi and active vitiligo, post-inflammatory hypopigmentation and stable vitiligo, and between stable and active vitiligo. In the analysis of dermoscopic features related to perilesional pigmentation, statistically significant differences (*p* < 0.05) were observed between post-inflammatory depigmentation and active/stable vitiligo, as well as between active and stable vitiligo (Table 2).

**Table 2:** Results of two-by-two comparisons of the chi-square test for clinical information and dermatoscopic features for all six diseases.

Disease category and percentage	Location		*χ*²	*p*-value	Distinct margins		χ²	*p*-value	Telangiectasia		χ²	*p*-value	Perilesional/marginal hyperpigmentation		χ²	*p*-value
	Head, face, and neck	Trunk and limbs			No	Yes			Lack	Exist			Lack	Exist		
	*N* = 253	*N* = 205			*N* = 278	*N* = 97			*N* = 118	*N* = 340			*N* = 127	*N* = 138		
Pityriasis alba	98_a_	1_a_	108.269	0.000^a^					17_a_	82_a_	50.818	0.000^a^				
Percentage	38.70	0.50							14.40	24.10						
Nevus depigmentosus	29_b_	65_b_			83_a_	11_a_	159.4	0.000^a^	20_a_	74_a_						
Percentage	11.50	31.70			29.90	11.30			16.90	21.80						
Post-inflammatory hypopigmentation	23_bc_	19_bc_			36_a_	6_a_			19_b_	23_b_			25_a_	17_a_	128.1	0.000^a^
Percentage	9.10	9.30			12.90	6.20			16.10	6.80			19.70	12.30		
Stable vitiligo	56_c_	55_c_			34_b_	77_b_			11_a_	100_a_			9_b_	102_b_		
Percentage	22.10	26.80			12.20	79.40			9.30	29.40			7.10	73.90		
Active vitiligo	47_bc_	65_bc_			110_c_	2_c_			51_b_	61_b_			93_c_	19_c_		
Percentage	18.60	31.70			39.60	2.10			43.20	17.90			73.20	13.80		
Lichen sclerosus					15_ac_	1_ac_										
Percentage					5.40%	1										

Each subscript letter represents a subset of each disease category, columns with the same subscript letter indicate no statistical difference, e.g., in the comparison of “Localization” (Columns 2–3), *pityriasis alba* is labeled with the subscript letter a, which differs from the subscripts b, bc, or c assigned to other conditions, indicating statistically significant differences between *pityriasis alba* and the other diseases. In contrast, *post-inflammatory hypopigmentation* (subscript bc) shares subscript letters with *nevus depigmentosus* (b) and *stable vitiligo* (c), demonstrating no statistically significant differences between *post-inflammatory hypopigmentation* and these two conditions.

^a^ Pearson’s chi-square test.

### 3.3 Dermatologists' performance on two tests

**Figure 3:**
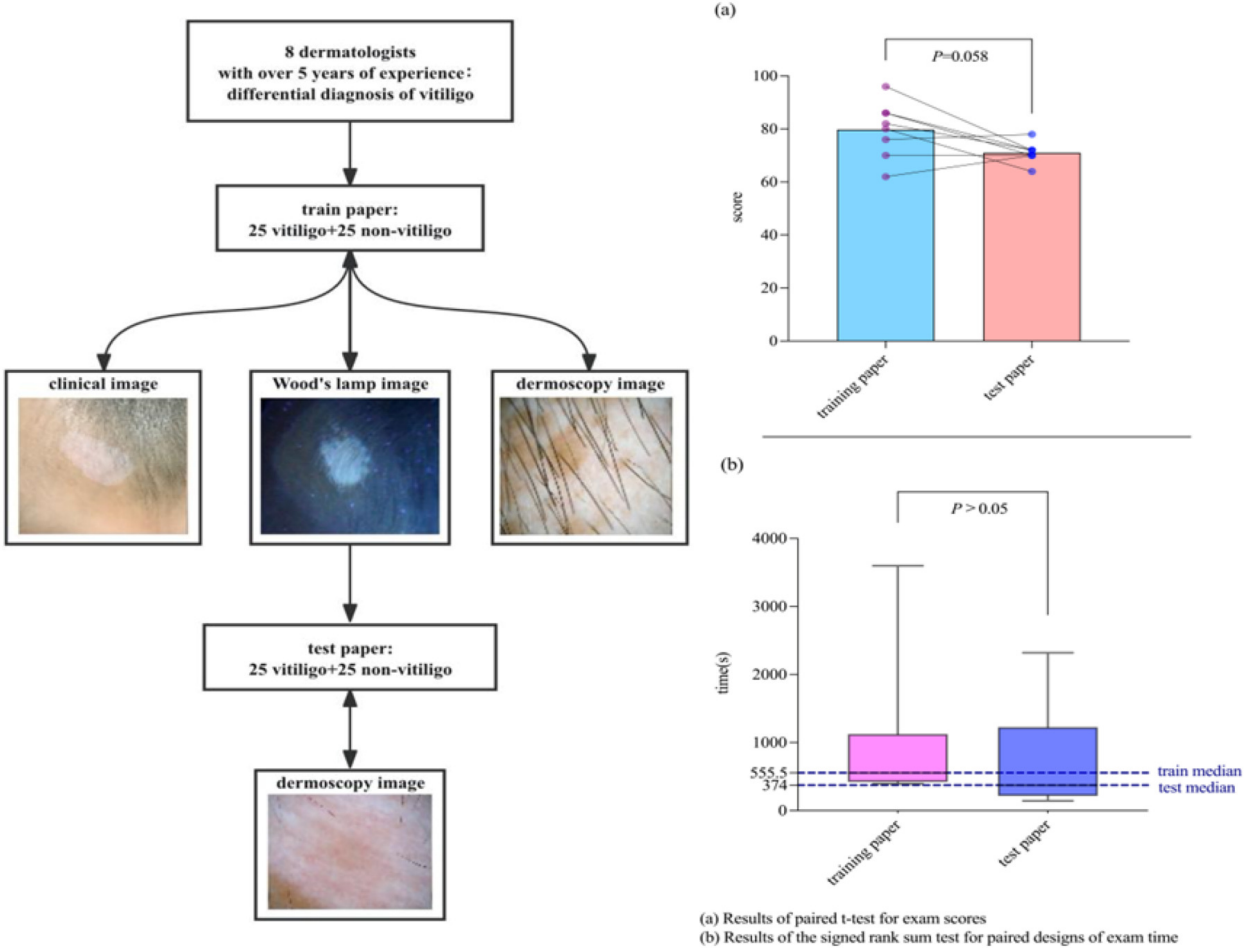
Comparison of the combination of photographs, Wood's lamp images, dermoscopic images (control group) vs. dermoscopic images only when vitiligo is diagnosed by dermatologists. (**a**) Results of paired *t*-test for exam scores. (**b**) Results of the signed rank sum test for paired designs of exam time.

The results showed that there were no statistically significant differences in the average scores (79.75 vs. 71; *p* = 0.058) and median duration (555.50 vs. 374 s; *p* = 1.000) between the training and test papers completed by the eight dermatologists (Figure 3). In addition, subgroup analysis comparing physicians with more than 8 years of clinical experience to those with 8 years or less revealed no statistically significant differences in mean diagnostic scores or median time spent on either test (*p* > 0.05).

### 3.4 Correlation between clinical and dermoscopic features

Analysis of clinical data and dermatoscopic features revealed several statistically significant associations. In patients with active vitiligo, disease duration was significantly associated with intralesional erythema [*p* = 0.031, odds ratio (OR) = 8.973, 95% confidence interval (CI) 1.219–66.067]. Significant associations were observed between lesion location (trunk: OR 3.288, 95% CI 1.312–8.237, *p* = 0.011; limbs: OR 5.959, 95% CI 1.554–22.853, *p* = 0.009; multiple sites: OR 4. 329, 95% CI 1.306–14.347, *p* = 0.017) and intralesional red dots/globules, using head/face/neck as reference and inverted pigment network [*p* = 0.004, OR = 4.347, 95% CI (1.593–11.864); *p* = 0.003, OR = 7.736, 95% CI (1.998–29.955); *p* = 0.036, OR = 3.818, 95% CI (1.095–13.311)]. In stable vitiligo, location (trunk vs. head, face, and neck) was significantly associated with dermoscopic features such as the reverse pigmentation network (*p* = 0.035, OR = 2.691, 95% CI 1.070–6.766) and perifollicular pigmentation (*p* = 0.044, OR = 2.650, 95% CI 1.027–6.840) (see Figures S2, S3 in the Supplementary Material). No statistically significant differences were found between gender, location, and dermatoscopic features in pityriasis alba and depigmented nevi. However, in post-inflammatory hypopigmentation, there were statistically significant differences between lesion location (trunk and limbs vs. head, face, and neck) and the gray network pattern (*p* = 0.011, OR = 19.503, 95% CI 1.985–191.606; *p* = 0.045, OR = 5.279, 95% CI (1.039–26.821) (see Table S3 in the Supplementary Material).

## 4 Discussion

Dermoscopy is a commonly used, simple, and non-invasive diagnostic tool with standardized terminology and basic parameters (33). It allows for detailed observation of pigmentation and vascular structures within lesion areas (34) and plays an important role in both the differential diagnosis and stage assessment of vitiligo (28).

This study found significant differences in the distribution of multiple dermoscopic features between active and stable vitiligo, both across different patients and within the same patient. Distinct margins, intralesional erythema, perifollicular pigmentation, telangiectasia, intralesional pigmentation isles, and perilesional/marginal hyperpigmentation were often observed dermoscopically in typical stable vitiligo. In contrast, features such as the trichrome pattern, micro-Koebner phenomenon, comet tail pattern, chalky white background, and starburst pattern—consistent with previous reports—were more frequently seen in active vitiligo (35, 36). Among these, perilesional/marginal hyperpigmentation, intralesional erythema, and intralesional pigmentation isles demonstrated higher diagnostic efficacy for stable or regressive vitiligo, whereas the micro-Koebner phenomenon/comet tail pattern and trichrome sign showed higher diagnostic efficacy in active vitiligo. These findings demonstrated that dermoscopic features vary with disease stage and treatment progress, highlighting the utility of dermoscopy in monitoring therapeutic response (34, 37).

Previous studies have reported the significance of dermatoscopy in the differential diagnosis of vitiligo (31). This study revealed the value of dermatoscopy in distinguishing vitiligo from pityriasis alba, depigmented nevi, sclerosing atrophic moss, and post-inflammatory hypopigmentation. We identified disease-specific dermoscopic patterns and observed statistically significant differences in common features when comparing various stages of vitiligo with other hypopigmentary disorders. For example, stable vitiligo was more likely to exhibit distinct margins, perilesional/marginal hyperpigmentation, and telangiectasia, whereas active vitiligo was more frequently associated with poorly defined borders. These distinctive features facilitate the differential diagnosis of several diseases, complementing previous research findings (30, 38).

Clinical analysis revealed that dermoscopy alone showed comparable diagnostic accuracy for vitiligo when compared with combined imaging methods (clinical photographs, Wood's lamp, and dermoscopy). In other words, dermatoscopy could be used as a standalone tool for the differential diagnosis of vitiligo, even in the absence of clinical data such as photographs and Wood's lamp images (39). This finding inspired us to explore the integration of artificial intelligence with current dermoscopy technology (40). A novel AI-powered dermoscopy system may be developed to detect characteristic dermoscopic features, enabling automated vitiligo diagnosis and assessment (41). Such a system could reduce labor and time costs in routine clinical practice, as well as improve diagnostic accuracy and treatment efficacy (42).

Our study suggests that telangiectasia is a clinically significant dermoscopic feature in active vitiligo persisting beyond 6 months. The inverted pigment network should focus more on lesions located outside the head, face, and neck regions or those with lower VIDA scores. Intralesional erythema, indicated by dilated capillaries (43), may suggest possible inflammation in the affected area. A prolonged active phase is more likely to exhibit inflammatory responses, and intralesional erythema may reflect the chronic nature of the disease and is associated with complex pathogenesis, such as immunity and oxidative stress (44). The inverted pigment network, characteristic of active vitiligo, results from pigment loss in skin grooves with partial retention of pigment in the skin ridges, indicating a process of pigment loss (45, 46). In stable vitiligo, truncal lesions require particular attention to the inverted pigment network and perifollicular pigmentation, which are associated with regional variations in melanin distribution patterns (47, 48). These dermoscopic patterns further elucidate the clinical-dermoscopic correlations in pediatric active vitiligo (49, 50), encouraging further exploration into the utility of multimodal data, including clinical and imaging data, for vitiligo assessment.

## 5 Conclusion

Dermoscopy presents different characteristics at different stages of vitiligo and across other hypopigmentation diseases. Therefore, it is a valuable tool for both diagnosis and staging in the pediatric population.

This study includes a relatively large sample size with regional representativity and focuses on analyzing dermoscopic features of vitiligo and common hypopigmentary disorders in children and adolescents. These findings contribute to improved diagnostic accuracy in early-stage vitiligo and hold significant clinical relevance for practical application. However, this study was conducted at a single research center and limited to participants aged 0–18 years, which may reduce the generalizability of the results. Additionally, the evaluation of clinical utility in this study was limited to manual assessment. Future research should be conducted at multiple centers with larger samples of a wider age range and additional diseases. Further exploration into AI-optimized dermoscopic protocols for vitiligo (51) is also warranted to enhance clinical utility.

## Data Availability

The original contributions presented in the study are included in the article/**Supplementary Material**, further inquiries can be directed to the corresponding author/s.

## References

[1] LeWitt TM Kundu RV. Vitiligo. JAMA Dermatol. (2021) 157(9):1136. 10.1001/jamadermatol.2021.1688 34287629

[2] Dabas G Vinay K Parsad D Kumar A Kumaran MS. Psychological disturbances in patients with pigmentary disorders: a cross-sectional study. J Eur Acad Dermatol Venereol. (2020) 34(2):392–9. 10.1111/jdv.15987 31566833

[3] Diotallevi F Gioacchini H De Simoni E Marani A Candelora M Paolinelli M Vitiligo, from pathogenesis to therapeutic advances: state of the art. Int J Mol Sci. (2023) 24(5):4910. 10.3390/ijms24054910 36902341 PMC10003418

[4] Nicolaidou E Mastraftsi S Tzanetakou V Rigopoulos D. Childhood vitiligo. Am J Clin Dermatol. (2019) 20(4):515–26. 10.1007/s40257-019-00430-0 30911977

[5] van Geel N Speeckaert R Taieb A Ezzedine K Lim HW Pandya AG Worldwide expert recommendations for the diagnosis and management of vitiligo: position statement from the International Vitiligo Task Force part 1: towards a new management algorithm. J Eur Acad Dermatol Venereol. (2023) 37(11):2173–84. 10.1111/jdv.19451 37746876

[6] Huang YW Arkesteijn W Lai YJ Ng CY. A comparative study of an advanced skin imaging system in diagnosing facial pigmentary and inflammatory conditions. Sci Rep. (2024) 14(1):14673. 10.1038/s41598-024-63274-7 38918427 PMC11199608

[7] Micali G Verzi AE Lacarrubba F. Alternative uses of dermoscopy in daily clinical practice: an update. J Am Acad Dermatol. (2018) 79(6):1117–32.e1. 10.1016/j.jaad.2018.06.021 29920317

[8] Kumar Jha A Sonthalia S Lallas A Chaudhary RKP. Dermoscopy in vitiligo: diagnosis and beyond. Int J Dermatol. (2018) 57(1):50–4. 10.1111/ijd.13795 29076154

[9] Navarrete-Dechent C Jaimes N Marchetti MA. Unveiling melanomagenesis through the dermatoscope. J Eur Acad Dermatol Venereol. (2021) 35(5):1038–9. 10.1111/jdv.17244 33885196

[10] Conforti C Giuffrida R Agozzino M Cannavo PS Dianzani C di Meo N Basal cell carcinoma and dermal nevi of the face: comparison of localization and dermatoscopic features. Int J Dermatol. (2021) 60(8):996–1002. 10.1111/ijd.15554 33825193

[11] Sun Y Su L Zhang Y Wang Z Chen S Gu H Dermatoscopic features differentiating among port wine stain, arteriovenous malformation, and capillary malformation-arteriovenous malformation syndrome: to detect potential fast-flow vascular malformations at an early stage. J Am Acad Dermatol. (2022) 87(6):1435–7. 10.1016/j.jaad.2022.07.053 35952834

[12] Yuan M Xie Y Zheng Y Zhang Z Yang C Li J. Novel ultraviolet-dermoscopy: early diagnosis and activity evaluation of vitiligo. Skin Res Technol. (2023) 29(1):e13249. 10.1111/srt.13249 36464842 PMC9838782

[13] Nayak D Vinay K Dev A Bishnoi A Kumaran MS Parsad D. Dermatoscopic evaluation of treatment response in patients undergoing NBUVB phototherapy for the treatment of non-segmental vitiligo: a prospective study. Photodermatol Photoimmunol Photomed. (2023) 39(3):279–81. 10.1111/phpp.12825 35953438

[14] Gupta P Dev A Vinay K Bishnoi A Kumaran MS Parsad D. Validating a novel technique of dermatoscopic imaging or sequential comparison of target lesions in vitiligo. Dermatol Pract Concept. (2024) 14(1):e2024003. 10.5826/dpc.1401a3 38364413 PMC10868769

[15] Delbaere L van Causenbroeck J Duponselle J Van Goethem C Speeckaert R van Geel N. Hot spots for clinical signs of disease activity in vitiligo. Exp Dermatol. (2024) 33(1):e14975. 10.1111/exd.14975 37975576

[16] Zhang L Chen S Kang Y Wang X Yan F Jiang M Association of clinical markers with disease progression in patients with vitiligo from China. JAMA Dermatol. (2020) 156(3):288–95. 10.1001/jamadermatol.2019.4483 31968061 PMC6990655

[17] van Geel N Grine L De Wispelaere P Mertens D Prinsen CAC Speeckaert R. Clinical visible signs of disease activity in vitiligo: a systematic review and meta-analysis. J Eur Acad Dermatol Venereol. (2019) 33(9):1667–75. 10.1111/jdv.15604 31131483

[18] Abdel-Wahab HM Ragaie MH. Pityriasis alba: toward an effective treatment. J Dermatolog Treat. (2022) 33(4):2285–9. 10.1080/09546634.2021.1959014 34289784

[19] Rao M Young K Jackson-Cowan L Kourosh A Theodosakis N. Post-inflammatory hypopigmentation: review of the etiology, clinical manifestations, and treatment options. J Clin Med. (2023) 12(3):1243. 10.3390/jcm12031243 36769891 PMC9917556

[20] Hudson A Sturgeon A Peiris A. Tinea versicolor. JAMA. (2018) 320(13):1396. 10.1001/jama.2018.12429 30285180

[21] Kraus CN. Vulvar lichen sclerosus. JAMA Dermatol. (2022) 158(9):1088. 10.1001/jamadermatol.2022.0359 35793083

[22] Powell JJ Wojnarowska F. Lichen sclerosus. Lancet. (1999) 353(9166):1777–83. 10.1016/S0140-6736(98)08228-2 10348006

[23] Kim SK Kang HY Lee ES Kim YC. Clinical and histopathologic characteristics of nevus depigmentosus. J Am Acad Dermatol. (2006) 55(3):423–8. 10.1016/j.jaad.2006.04.053 16908347

[24] Njoo MD Das PK Bos JD Westerhof W. Association of the Kobner phenomenon with disease activity and therapeutic responsiveness in vitiligo vulgaris. Arch Dermatol. (1999) 135(4):407–13. 10.1001/archderm.135.4.407 10206047

[25] Hamzavi I Jain H McLean D Shapiro J Zeng H Lui H. Parametric modeling of narrowband UV-B phototherapy for vitiligo using a novel quantitative tool: the vitiligo area scoring index. Arch Dermatol. (2004) 140(6):677–83. 10.1001/archderm.140.6.677 15210457

[26] Lewis-Jones MS Finlay AY. The Children’s Dermatology Life Quality Index (CDLQI): initial validation and practical use. Br J Dermatol. (1995) 132(6):942–9. 10.1111/j.1365-2133.1995.tb16953.x 7662573

[27] Kostopoulou P Jouary T Quintard B Ezzedine K Marques S Boutchnei S Objective vs. subjective factors in the psychological impact of vitiligo: the experience from a French referral centre. Br J Dermatol. (2009) 161(1):128–33. 10.1111/j.1365-2133.2009.09077.x 19298280

[28] Godinez-Chaparro JA Roldan-Marin R Vidaurri-de la Cruz H Soto-Mota LA Ferez K. Dermatoscopic patterns in vitiligo. Dermatol Pract Concept. (2023) 13(4):e2023197. 10.5826/dpc.1304a197 37992390 PMC10656144

[29] Godinez-Chaparro JA Roldan-Marin R Soto-Mota A Calzada-Mendoza CC. Dermatoscopic patterns in childhood vitiligo and their association with reflectance confocal microscopy findings. Dermatol Pract Concept. (2023) 13(4):e2023306. 10.5826/dpc.1304a306 37695810 PMC10656151

[30] Thomas IN James JJ Bala A Mohan S Dogiparthi S Shanmugam NP Sr. Usage of dermoscopy as an effective diagnostic tool in pityriasis alba: a prospective observational study among children in a suburban hospital in south India. Cureus. (2023) 15(6):e40271. 10.7759/cureus.40271 37448397 PMC10336270

[31] Al-Refu K. Dermoscopy is a new diagnostic tool in diagnosis of common hypopigmented macular disease: a descriptive study. Dermatol Rep. (2019) 11(1):7916. 10.4081/dr.2018.7916 PMC6509478 31119026

[32] Errichetti E Stinco G. Dermoscopy in general dermatology: a practical overview. Dermatol Ther. (2016) 6(4):471–507. 10.1007/s13555-016-0141-6 PMC5120630 27613297

[33] Errichetti E Zalaudek I Kittler H Apalla Z Argenziano G Bakos R Standardization of dermoscopic terminology and basic dermoscopic parameters to evaluate in general dermatology (non-neoplastic dermatoses): an expert consensus on behalf of the International Dermoscopy Society. Br J Dermatol. (2020) 182(2):454–67. 10.1111/bjd.18125 31077336

[34] Ring C Cox N Lee JB. Dermatoscopy. Clin Dermatol. (2021) 39(4):635–42. 10.1016/j.clindermatol.2021.03.009 34809768

[35] Gupta P Vinay K Bishnoi A Kumaran MS Parsad D. A prospective observational study to sequentially determine the dermoscopic features of vitiligo and its association with disease activity in patients on medical treatment: dermoscopy and disease activity in vitiligo. Pigment Cell Melanoma Res. (2023) 36(1):33–41. 10.1111/pcmr.13069 36112075

[36] Thatte SS Khopkar US. The utility of dermoscopy in the diagnosis of evolving lesions of vitiligo. Indian J Dermatol Venereol Leprol. (2014) 80(6):505–8. 10.4103/0378-6323.144144 25382506

[37] Ibrahim S Hegazy RA Gawdat HI Esmat S Mahmoud E Rashed L Differentiating active from stable vitiligo: the role of dermoscopic findings and their relation to CXCL10. J Cosmet Dermatol. (2022) 21(10):4651–8. 10.1111/jocd.14922 35298096

[38] Roh D Shin K Kim WI Yang MY Lee WK Kim GW Clinical differences between segmental nevus depigmentosus and segmental vitiligo. J Dermatol. (2019) 46(9):777–81. 10.1111/1346-8138.15015 31342527

[39] Abdi P Anthony MR Farkouh C Chan AR Kooner A Qureshi S Non-invasive skin measurement methods and diagnostics for vitiligo: a systematic review. Front Med. (2023) 10:1200963. 10.3389/fmed.2023.1200963 PMC10416110 37575985

[40] Li Pomi F Papa V Borgia F Vaccaro M Pioggia G Gangemi S. Artificial intelligence: a snapshot of its application in chronic inflammatory and autoimmune skin diseases. Life. (2024) 14(4):516. 10.3390/life14040516 38672786 PMC11051135

[41] Du-Harpur X Watt FM Luscombe NM Lynch MD. What is AI? Applications of artificial intelligence to dermatology. Br J Dermatol. (2020) 183(3):423–30. 10.1111/bjd.18880 31960407 PMC7497072

[42] Li Y Kong AW Thng S. Segmenting vitiligo on clinical face images using CNN trained on synthetic and internet images. IEEE J Biomed Health Inform. (2021) 25(8):3082–93. 10.1109/JBHI.2021.3055213 33513120

[43] Kittler H Marghoob AA Argenziano G Carrera C Curiel-Lewandrowski C Hofmann-Wellenhof R Standardization of terminology in dermoscopy/dermatoscopy: results of the third consensus conference of the international society of dermoscopy. J Am Acad Dermatol. (2016) 74(6):1093–106. 10.1016/j.jaad.2015.12.038 26896294 PMC5551974

[44] Abdel-Malek ZA Jordan C Ho T Upadhyay PR Fleischer A Hamzavi I. The enigma and challenges of vitiligo pathophysiology and treatment. Pigment Cell Melanoma Res. (2020) 33(6):778–87. 10.1111/pcmr.12878 32198977

[45] Thatte SS Dongre AM Khopkar US. “Reversed pigmentary network pattern” in evolving lesions of vitiligo. Indian Dermatol Online J. (2015) 6(3):222–3. 10.4103/2229-5178.156427 26009725 PMC4439759

[46] Nirmal B Antonisamy B Peter CVD George L George AA Dinesh GM. Cross-sectional study of dermatoscopic findings in relation to activity in vitiligo: BPLeFoSK criteria for stability. J Cutan Aesthet Surg. (2019) 12(1):36–41. 10.4103/JCAS.JCAS_75_18 31057267 PMC6484572

[47] Li QX Swanson DL Tu P Yang SX Li H. Clinical and dermoscopic features of surgically treated melanocytic nevi: a retrospective study of 1046 cases. Chin Med J. (2019) 132(17):2027–32. 10.1097/CM9.0000000000000416 31460902 PMC6793791

[48] Longo C Pampena R Moscarella E Chester J Starace M Cinotti E Dermoscopy of melanoma according to different body sites: head and neck, trunk, limbs, nail, mucosal and acral. J Eur Acad Dermatol Venereol. (2023) 37(9):1718–30. 10.1111/jdv.19221 37210653

[49] Jha AK Sonthalia S Lallas A. Dermoscopy as an evolving tool to assess vitiligo activity. J Am Acad Dermatol. (2018) 78(5):1017–9. 10.1016/j.jaad.2017.12.009 29229577

[50] van Geel N Speeckaert R De Wolf J Bracke S Chevolet I Brochez L Clinical significance of Koebner phenomenon in vitiligo. Br J Dermatol. (2012) 167(5):1017–24. 10.1111/j.1365-2133.2012.11111.x 22950415

[51] Li Y Thng STG Kong AW. Bridging the gap between vitiligo segmentation and clinical scores. IEEE J Biomed Health Inform. (2024) 28(3):1623–34. 10.1109/JBHI.2023.3342069 38100337

