# Supplementary material for: Dermoscopic features in children with vitiligo and other hypopigmentation disorders
Source: Front Pediatr. 2025 Jul 10;13:1550349. doi: 10.3389/fped.2025.1550349 (PMC12287109; doi:10.3389/fped.2025.1550349)
Supplement: Supplementary file 1 [file Datasheet1.docx]

**Supplementary materials：**

**
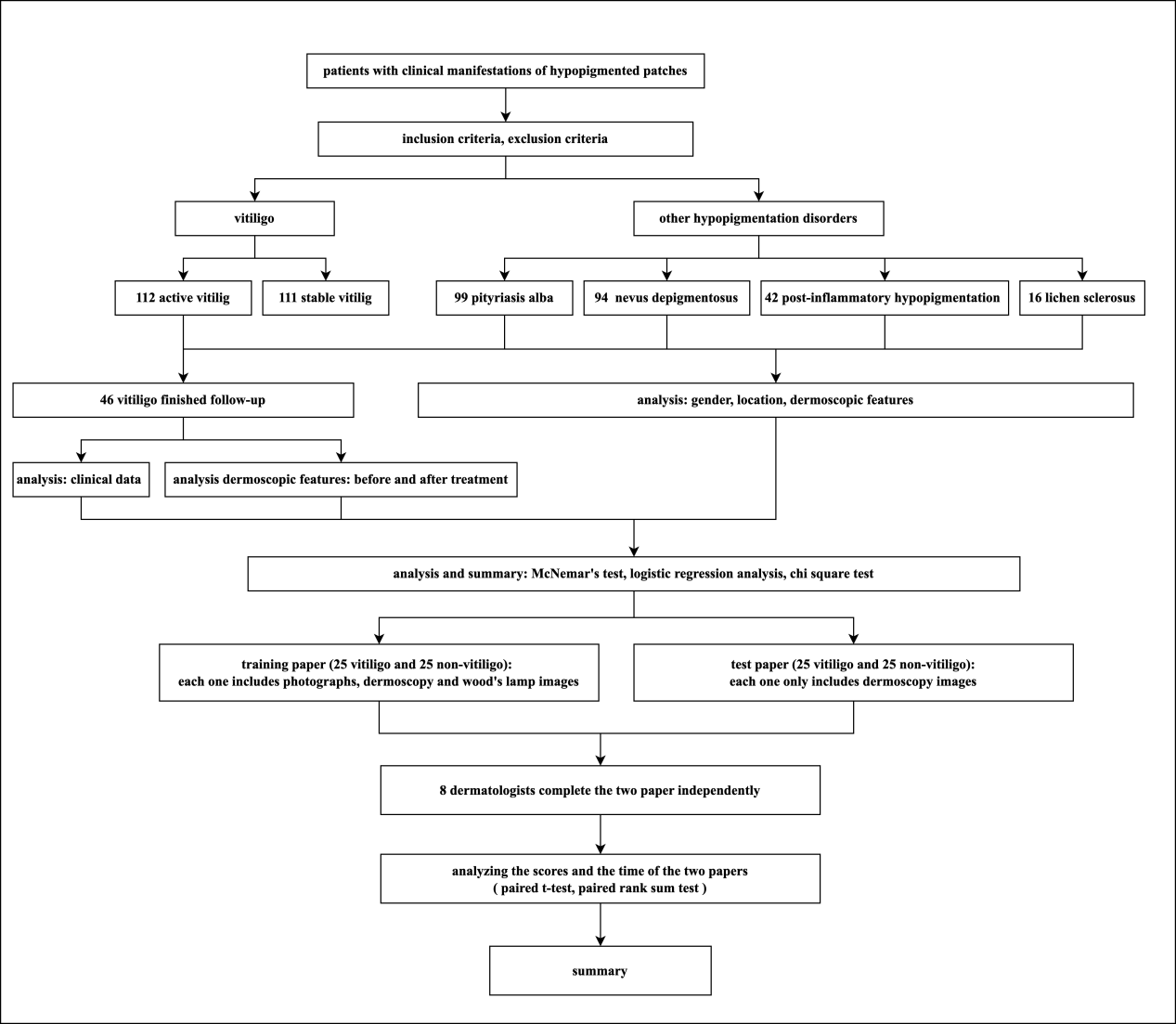
**

Figure 1 Technology roadmap.

| table 1 Clinical data analysis of 46 children with active vitiligo | | | | |
| --- | --- | --- | --- | --- |
| general information | category | N（%） | x±s | M（IQR） |
| gender | male | 23（50%） |  |  |
|  | female | 23（50%） |  |  |
| location | head, face, and neck | 20（43.5%） |  |  |
|  | trunk | 8（17.4%） |  |  |
|  | limbs | 5（10.8%） |  |  |
|  | ≥2 region | 13（28.3%） |  |  |
| Age (month) | ≥84 months | 29（63%） | 80.11±34.13 |  |
|  | ＞84 months | 17（37%） |  |  |
| disease duration (day) | ≤180 days | 21（45.7%） |  | 210（433） |
|  | ＞180 days | 25（54.3%） |  |  |
| classification | segmental | 12（26.1%） |  |  |
|  | non-segmental | 34（73.9%） |  |  |
| season of onset | from January to June | 28（60.9%） |  |  |
|  | from July to December | 18（39.1%） |  |  |
| trauma or exposure history | yes | 13（28.3%） |  |  |
|  | no | 33（71.7%） |  |  |
| Family history of first degree relatives (atopic disease） | yes | 17（37.0%） |  |  |
|  | no | 29（63.0%） |  |  |
| comorbidities (inflammatory or immune disorders) | yes | 15（32.6%） |  |  |
|  | no | 31（67.4%） |  |  |
| leukotrichia | yes | 18（39.1%） |  |  |
|  | no | 28（60.9%） |  |  |
| treatment | topical application | 8（17.3%） |  |  |
|  | topical and oral glucocorticoids | 11（24%） |  |  |
|  | topical medication and phototherapy | 7（15.2%） |  |  |
|  | topical and oral glucocorticoids, phototherapy | 20（43.5%） |  |  |
| repigmentation pattern | perifollicular type | 2（4.3%） |  |  |
|  | peri-lesion type | 9（19.6%） |  |  |
|  | diffuse type | 3（6.5%） |  |  |
|  | hybrid type | 32（69.5%） |  |  |
| oral dosage of glucocorticoids |  |  |  | 0.29（0.36） |
| BSA |  |  |  | 0.3（0.79） |
| VIDA |  |  |  | 3（2） |
| CDLQI |  |  |  | 2（4） |
| VASI |  |  |  | 0.3（0.79） |
| VAS |  |  |  | 2（5） |
| BSA, affected area of skin lesions; VIDA, vitiligo disease activity score; CDLQI, Children’s Dermatology Life Quality Index; VASI, vitiligo area score index; VAS, visual analogue scale | | | | |

| Table 2 Results of McNemar's test for dermoscopic features in 46 cases of active vitiligo before and after treatment | | | | | |
| --- | --- | --- | --- | --- | --- |
| dermoscopic features | variable | total (case) | no（%） | yes（%） | *P* value |
| intralesional erythema | pre-treatment | 46 | 31（67.4%） | 15（32.6%） | 0.000 |
|  | post-treatment | 46 | 2（4.3%） | 44（95.7%） |  |
| chalky white background | pre-treatment | 46 | 6（13.0%） | 40（87.0%） | 0.000 |
|  | post-treatment | 46 | 25（54.3%） | 21（45.7%） |  |
| inverted pigment network | pre-treatment | 46 | 16（34.8%） | 30（65.2%） | 0.022 |
|  | post-treatment | 46 | 7（15.2%） | 39（84.8%） |  |
| perifollicular pigmentation | pre-treatment | 46 | 25（54.3%） | 21（45.7%） | 0.000 |
|  | post-treatment | 46 | 8（17.4%） | 38（82.6%） |  |
| telangiectasia | pre-treatment | 46 | 8（17.4%） | 38（82.6%） | 0.016 |
|  | post-treatment | 46 | 1（2.2%） | 45（97.8%） |  |
| intralesional repigmentation isles | pre-treatment | 46 | 40（87.0%） | 6（13.0%） | 0.000 |
|  | post-treatment | 46 | 15（32.6%） | 31（67.4%） |  |
| tapioca sago appearance | pre-treatment | 46 | 38（82.6%） | 8（17.4%） | 0.012 |
|  | post-treatment | 46 | 29（63.0%） | 17（37.0%） |  |
| micro-Koebner or comet tail pattern | pre-treatment | 46 | 20（43.5%） | 26（56.5%） | 0.000 |
|  | post-treatment | 46 | 45（97.8%） | 1（2.2%） |  |
| leukotrichia | pre-treatment | 46 | 12（26.1%） | 34（73.9%） | 1.000 |
|  | post-treatment | 46 | 11（23.9%） | 35（76.1%） |  |
| starburst pattern | pre-treatment | 46 | 39（84.8%） | 7（15.2%） | 0.070 |
|  | post-treatment | 46 | 45（97.8%） | 1（2.2%） |  |
| intralesional red dots or globules | pre-treatment | 46 | 29（63.0%） | 17（37.0%） | 0.774 |
|  | post-treatment | 46 | 31（67.4%） | 15（32.6%） |  |
| distinct margins | pre-treatment | 46 | 46（100%） | 0 | n.a. |
|  | post-treatment | 46 | 3（6.5%） | 43（93.5%） |  |
| reduced or absent pigment network | pre-treatment | 46 | 0 | 46（100%） | n.a. |
|  | post-treatment | 46 | 6（13.0%） | 40（87.0%） |  |
| perifollicular depigmentation | pre-treatment | 46 | 0 | 46（100%） | n.a. |
|  | post-treatment | 46 | 0 | 46（100%） |  |
| trichromic pattern | pre-treatment | 46 | 5（10.9%） | 41（89.1%） | n.a. |
|  | post-treatment | 46 | 46（100%） | 0 |  |
| perilesional/marginal hyperpigmentation | pre-treatment | 46 | 40（87.0%） | 6（13.0%） | n.a. |
|  | post-treatment | 46 | 0 | 46（100%） |  |
| n.a., not assessable. |  |  |  |  |  |





Figure 2 Logistic regression analysis (a) gender, location, and dermoscopic features (intralesional red dots or globules) in 112 cases of active vitiligo, (b) gender, location, and dermoscopic features (inverted pigment network) in 112 cases of active vitiligo, (c) gender, location, and dermoscopic features (inverted pigment network) in 111 cases of stable vitiligo, (d) gender, location, and dermoscopic features (Perifollicular pigmentation) in 111 cases of stable vitiligo

**
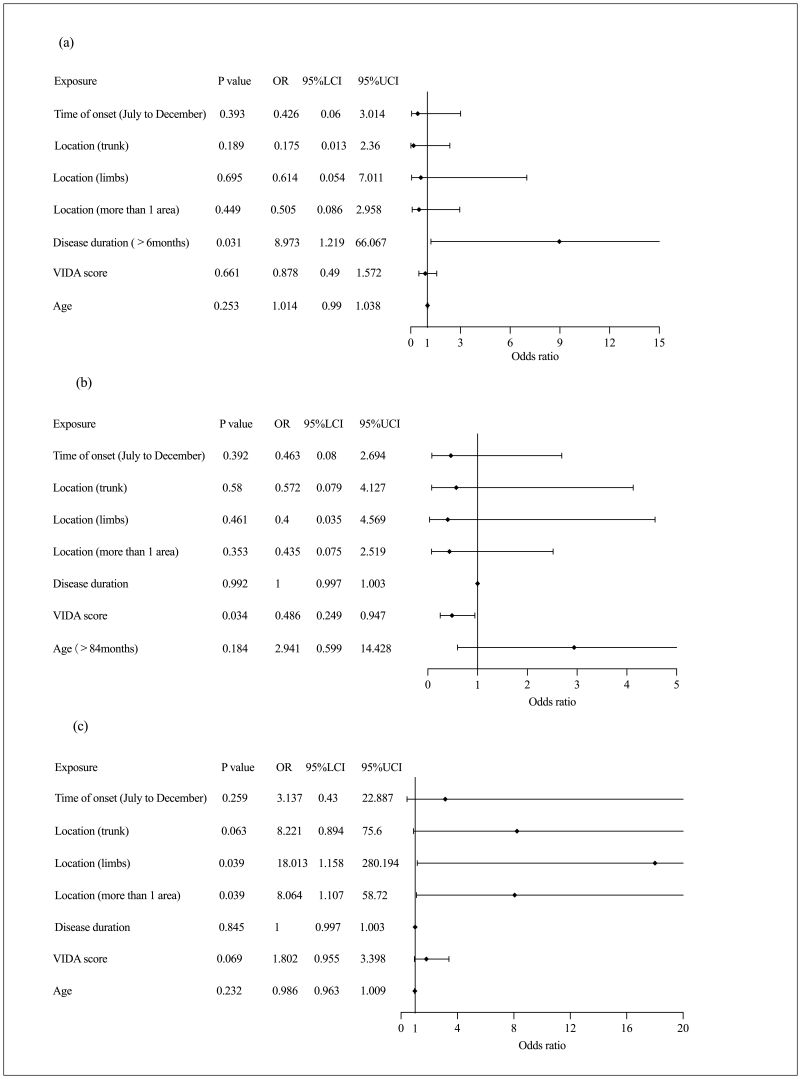
**

Figure 3 Logistic regression analysis of  46 cases of active vitiligo before treatment, (a) age, course, location, VIDA score, onset season, and dermoscopic features (intralesional erythema), (b) age, course, location, VIDA score, onset season, and dermoscopic features (inverted pigment network), (c) age, course, location, VIDA score, onset season, and dermoscopic features (intralesional red dots or globules)

| table3 Logistic analysis of gender, location and gray network in 42 cases of post-inflammatory hypopigmentation | | | | | | | |
| --- | --- | --- | --- | --- | --- | --- | --- |
| variable | B value | SE value | Wald value | P value | OR value | 95%CI of OR | |
| constant | -1.428 | 1.164 | 1.504 | 0.22 | 0.24 |  |  |
| gender^a^ | 0.413 | 0.727 | 0.323 | 0.57 | 1.512 | 0.364 | 6.285 |
| trunk^b^ | 2.971 | 1.166 | 6.493 | 0.011 | 19.503 | 1.985 | 191.606 |
| limbs^b^ | 1.664 | 0.829 | 4.024 | 0.045 | 5.279 | 1.039 | 26.821 |
| a, compared to male; b, compared to head, face and neck | | | | | | | |

**Description of dermatoscopic structures in Vitiligo：**

| **Pattern/signs** | **Description** |
| --- | --- |
| Perifollicular pigmentation | Achromic spots at the pilosebaceous orifice present a homogeneous surrounding pigment with a tone like that of normal skin. |
| Reduced or absent pigment network | Whitening or loss of the pigment network that causes an absent pigment network. |
| Inverted pigment network | The white or depigmented pattern is in the form of a network with intermediate pigmentation. |
| Trichromic | Depigmented middle area of pseudo-scarring appearance, surrounding the main area is a faint yellowish-brown pigmentation and in the periphery the characteristic phototype of the patient (3 different shades). |
| Starburst | Extension or peripheral whitish linear projections in various directions. |
| Comet tail | Unidirectional linear extension or projection to an area adjacent to the initial vitiligo lesion. |
| micro-Koebner phenomenon | The appearance of isomorphic depigmented linear stretch marks distributed along the trauma line or around the main patch of vitiligo. |
| Tapioca sago or satellite lesions | Small white spots with no structure less than 1 mm in diameter are located around the main patch of vitiligo right on the perilesional skin that looks clinically normal. |
| Leukotrichia | White hairy hairs |
| Perifollicular depigmentation | Absence of surrounding pigment in the pilosebaceous orifice. |
| Perilesional/marginal hyperpigmentation | Darker pigmentation around achromic lesions. |
| Intra/perilesional erythema | Redness of the skin depigmented or on its margins. |
| Telangiectasias | Bright red dilated capillaries 1 to 4 mm in diameter; resembled spider veins. |
| Atrophy | The achromic spot that presents a decrease of one or several layers of the skin and skin annexes, causing thinned, folded skin , accompanied by telangiectasias. |
| Perifollicular pigmentation | The pilosebaceous orifice in the depigmented areas which has homogeneous surrounding pigment and is darker than that of normal skin. |

**references**

1. Godínez-Chaparro JA, Roldán-Marín R, Vidaurri-de la Cruz H, Soto-Mota LA, Férez K. Dermatoscopic Patterns in Vitiligo. Dermatol Pract Concept. 2023 Oct 1;13(4):e2023197. doi: 10.5826/dpc.1304a197. PMID: 37992390; PMCID: PMC10656144.
2. Godinez-Chaparro JA, Roldan-Marin R, Soto-Mota A, Calzada-Mendoza CC. Dermatoscopic Patterns in Childhood Vitiligo and Their Association With Reflectance Confocal Microscopy Findings. Dermatol Pract Concept. 2023 Oct 1;13(4).

**Description of dermatoscopic structures in the other hypopigmentation diseases**：

| **Pattern/signs** | **Description** |
| --- | --- |
| scattered small pearly white globules | Small white spots with no structure less than 1 mm in diameter which are located within the patch |
| follicular keratin plugs | White-yellowish structureless areas, white chrysalis like structures. |
| perilesional erythema | Redness of the skin on its margins. |
| tiny grayish white scales | The fairly ill-demarcated white area with fine scales that are commonly distributed within and outside the macules. |
| pale hypopigmentation spots | The hypopigmented macules which are fairly ill-demarcated white area |
| serrated edges | Sharp margin to differentiate the hypopigmented patch from the surrounding skin. |
| scar-like white structures | white structureless areas with telangiectasia of different lengths and calibers |
| acne-like openings | comedolike openings |
| reticular distribution | The borders showing pseudopods pattern protruding into the normal skin. |
| gray network | Patches showing faint depigmented reticular network within them. |
| irregular morphology | The depigmented patches which do not have a fixed morphological distribution |
| dotted or reticular pigmentation | Pigmented spots with small dot or net-like distribution, observed in the white structureless areas |
| thin or atrophic epidermis | Skin grooves and ridges which cannot be seen in the white structureless area |

**references**

[1] Thomas IN, James JJ, Bala A, Mohan S, Dogiparthi S, Shanmugam NP, Sr. Usage of Dermoscopy as an Effective Diagnostic Tool in Pityriasis Alba: A Prospective Observational Study Among Children in a Suburban Hospital in South India. Cureus. 2023 Jun;15(6):e40271.

[2] Al-Refu K. Dermoscopy is a new diagnostic tool in diagnosis of common hypopigmented macular disease: A descriptive study. Dermatol Reports. 2019 Jan 23;11(1):7916.

[3] Errichetti E, Stinco G. Dermoscopy in General Dermatology: A Practical Overview. Dermatol Ther (Heidelb). 2016 Dec;6(4):471-507.
